# Deqi Sensation in Different Kinds of Acupuncture 2014

**DOI:** 10.1155/2015/306138

**Published:** 2015-04-16

**Authors:** Cun-Zhi Liu, Gerhard Litscher, Fan-Rong Liang, Jian Kong, Lin-Peng Wang, Lu Wang

**Affiliations:** ^1^Acupuncture and Moxibustion Department, Beijing Hospital of Traditional Chinese Medicine Affiliated to Capital Medical University, 23 Meishuguanhou Street, Dongcheng District, Beijing 100010, China; ^2^Research Unit for Complementary and Integrative Laser Medicine, Research Unit of Biomedical Engineering in Anesthesia and Intensive Care Medicine, TCM Research Center Graz, Medical University of Graz, Auenbruggerplatz 29, 8036 Graz, Austria; ^3^College of Acupuncture and Massage, Chengdu University of Traditional Chinese Medicine, Chengdu, Sichuan 610075, China; ^4^Department of Psychiatry, Massachusetts General Hospital (MGH), Harvard Medical School, Charlestown, Boston, MA 02129, USA; ^5^School of Traditional Chinese Medicine, Capital Medical University, 10 Xitoutiao, Youanmen, Beijing 100069, China

This is an annual special issue published in this journal. The current issue is the 2014 issue which includes 7 interesting papers.

Acupuncture stimulation elicits Deqi, a composite of unique sensations that is essential for clinical efficacy according to traditional Chinese medicine. In recent years, clinical trials of acupuncture have paid increasing attention to the evocation of Deqi. The physiological mechanism that produces the effect of Deqi has also been explored in several studies but is not well understood.

This special issue contains 12 papers, of which 7 were published. Five papers are related to the characterization of the Deqi during acupuncture treatment. Y. Ren et al. conducted survey to determine acupuncturists' perspectives about Deqi. Deqi not only refers to needling sensations but also involves the changes of qi induced by needle insertion into the acupoint. Another 4 papers of clinical trials in patients with chronic fatigue syndrome, primary dysmenorrhea, depression, and motility of esophagus introduced the proper way to induce Deqi. These results may provide some evidences to the qualitative and quantitative research of Deqi.

Y.-S. Su et al. suggested somatosensory nerve fibers mediated generation of Deqi in manual acupuncture and local moxibustion-like stimuli-modulated gastric motility in rats. It is related to the physiological mechanism of Deqi. D.-S. Tian et al.'s paper is neuroimaging study on the interaction between Deqi and acupuncture. They revealed that acupuncture treatment with Deqi apparently increased acupoint blood flow, tissue displacement, and amplitude of myoelectricity and induced fMRI signal increase/decrease in different brain regions although no significant change was found in electroencephalography. This study provides evidence to understand neural mechanism underlying acupuncture.

Deqi should be taken into account in clinical trials, and more researches are required to understand the underlying mechanisms, as described in this special issue.

*Cun-Zhi Liu* *Cun-Zhi Liu* *Gerhard Litscher* *Gerhard Litscher* *Fan-Rong Liang* *Fan-Rong Liang* *Jian Kong* *Jian Kong* *Lin-Peng Wang* *Lin-Peng Wang* *Lu Wang* *Lu Wang*

